# Poly[aqua­hexa­benzimidazole­octa-μ-cyanido-octa­cyanidotricopper(II)ditungstate(V)]

**DOI:** 10.1107/S160053681000841X

**Published:** 2010-03-13

**Authors:** Kenta Imoto, Souhei Kaneko, Yoshihide Tsunobuchi, Koji Nakabayashi, Shin-ichi Ohkoshi

**Affiliations:** a Department of Chemistry, School of Science, University of Tokyo, 7-3-1 Hongo, Bunkyo-Ku, Tokyo 113-0033, Japan

## Abstract

In the polymeric title compound, [Cu_3_W_2_(CN)_16_(C_7_H_6_N_2_)_6_(H_2_O)]_*n*_, the coordination geometry of the W(V) atom is eight-coordinate dodeca­hedral, where four CN groups of [W(CN)_8_] are bridged to Cu^II^ ions, and the other four CN groups are not bridged. The coordination geometries of the Cu^II^ ions are five-coordinate pseudo-square-based pyramidal. There are two distinct Cu sites, which build and link the cyanido-bridged Cu—W ladder chains. Successive connections lead to the formation of a two-dimensional network. The H atoms of a coordinated water molecule and the imino groups form hydrogen bonds to the N atoms of non-bridged CN groups.

## Related literature

For general background to mol­ecule-based magnets, see: Catala *et al.* (2005 ▶); Garde *et al.* (1999 ▶); Herrera *et al.* (2004 ▶, 2008 ▶); Kosaka *et al.* (2009 ▶); Leipoldt *et al.* (1994 ▶); Ohkoshi *et al.* (2006 ▶, 2007 ▶, 2008 ▶); Sieklucka *et al.* (2009 ▶); Zhong *et al.* (2000 ▶). For related structures, see: Ohkoshi *et al.* (2003 ▶); Podgajny *et al.* (2002 ▶); Kaneko *et al.* (2007 ▶).
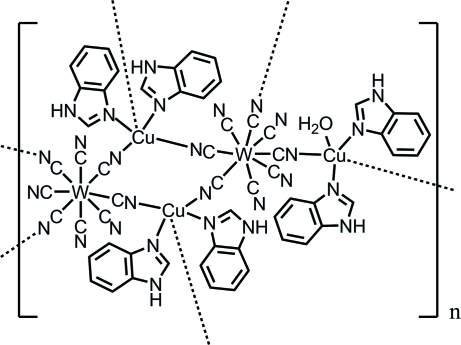


## Experimental

### 

#### Crystal data

[Cu_3_W_2_(CN)_16_(C_7_H_6_N_2_)_6_(H_2_O)]
*M* *_r_* = 1701.46
Monoclinic, 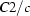

*a* = 32.0103 (8) Å
*b* = 10.2389 (3) Å
*c* = 19.5533 (5) Å
β = 93.8269 (8)°
*V* = 6394.3 (3) Å^3^
*Z* = 4
Mo *K*α radiation
μ = 4.63 mm^−1^
*T* = 296 K
0.45 × 0.12 × 0.08 mm

#### Data collection

Rigaku R-AXIS RAPID diffractometer
Absorption correction: multi-scan (*ABSCOR*; Higashi, 1995 ▶) *T*_min_ = 0.327, *T*_max_ = 0.690
31073 measured reflections
7321 independent reflections
6376 reflections with *I* > 2σ(*I*)
*R*_int_ = 0.059

#### Refinement

*R*[*F* ^2^ > 2σ(*F* ^2^)] = 0.030
*wR*(*F* ^2^) = 0.085
*S* = 1.02
7321 reflections
421 parameters
H atoms treated by a mixture of independent and constrained refinement
Δρ_max_ = 1.85 e Å^−3^
Δρ_min_ = −1.08 e Å^−3^

### 

Data collection: *PROCESS-AUTO* (Rigaku, 1998 ▶); cell refinement: *PROCESS-AUTO*; data reduction: *CrystalStructure* (Rigaku, 2007 ▶); program(s) used to solve structure: *SHELXS97* (Sheldrick, 2008 ▶); program(s) used to refine structure: *SHELXL97* (Sheldrick, 2008 ▶); molecular graphics: *ORTEP-3* (Farrugia, 1997 ▶) and *VESTA* (Momma & Izumi, 2006 ▶); software used to prepare material for publication: *CrystalStructure* .

## Supplementary Material

Crystal structure: contains datablocks I, global. DOI: 10.1107/S160053681000841X/pk2226sup1.cif

Structure factors: contains datablocks I. DOI: 10.1107/S160053681000841X/pk2226Isup2.hkl

Additional supplementary materials:  crystallographic information; 3D view; checkCIF report

## Figures and Tables

**Table 1:** Hydrogen-bond geometry (Å, °)

*D*—H⋯*A*	*D*—H	H⋯*A*	*D*⋯*A*	*D*—H⋯*A*
O1—H1⋯N6	1.05 (12)	2.20 (12)	3.178 (7)	154 (10)
N10—H10*N*⋯N5^i^	0.86	1.97	2.802 (5)	163
N12—H12*N*⋯N8^ii^	0.86	2.04	2.888 (5)	169
N14—H14*N*⋯N7^iii^	0.86	2.18	2.973 (5)	154

Symmetry codes: (i) 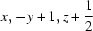; (ii) 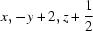; (iii) 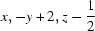.

**Fig. 1.**
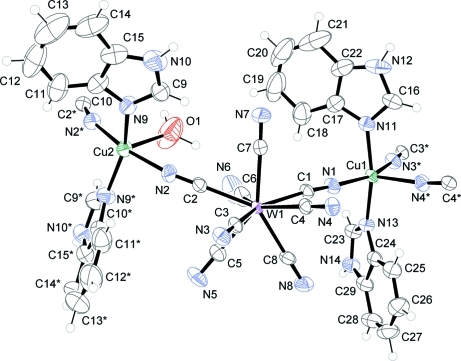
Thermal ellipsoid plots (50% probability level) of Cu3[W(CN)8]2(C7H6N2)6(H2O). Magenta, Green, gray, light blue, red, and white circle represent W, Cu, C, N, O, and H atoms, respectively. The asterisks indicate the atoms generated by symmetry operations.

**Fig. 2.**
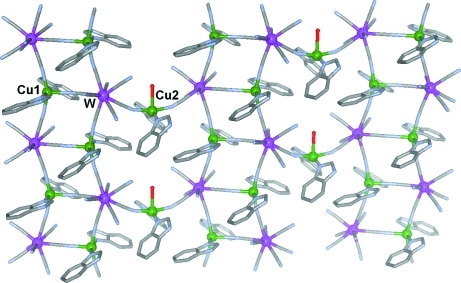
A structure diagram of Cu3[W(CN)8]2(C7H6N2)6(H2O). Magenta, Green, gray, light blue, and red represent W, Cu, C, N, and O atoms, respectively. Hydrogen atoms are omitted for clarity.

## supplementary crystallographic information

### Comment

In molecule-based magnets, preparing compounds with a high Curie temperature (*T*_C_) is a challenging issue. From this view point, octacyanometalate [*M*(CN)_8_] (*M* = Mo, W, Nb)-based magnets have been aggressively studied due to their high *T*_C_ (Garde *et al.*, 1999; Zhong *et al.*, 2000; Herrera *et al.*, 2008; Sieklucka *et al.*, 2009; Kosaka *et al.*, 2009) and properties such as photomagnetism (Herrera *et al.*, 2004; Catala *et al.*, 2005; Ohkoshi *et al.*, 2006; Ohkoshi *et al.*, 2008) and chemically sensitive magnetism (Ohkoshi *et al.*, 2007). Octacyanometalates, [*M*(CN)_8_] (*M* = Mo, W, Nb), a versatile class of building blocks, can adopt different spatial configurations depending on their chemical environment, *e.g.*, square antiprism (*D*_4d_), dodecahedron (*D*_2d_), and bicapped trigonal prism (*C*_2v_) (Leipoldt *et al.*, 1994). Thus, crystal structures of their complexes have various coordination geometries. Several octacyanometalate-based magnets of Cu—W systems such as {[Cu_3_[W(CN)_8_]_2_]3.4H_2_O}_n_ (3-dimensional network complex, 3-D) (Garde *et al.*, 1999), {[Cu_3_[*W*(CN)_8_]_2_(pyrimidine)_2_]8H_2_O}_n_ (3-D) (Ohkoshi *et al.*, 2007), {[(tetrenH_5_)_0.8_Cu_4_ [W(CN)_8_]_4_]7.2H_2_O}_n_ (2-D) (Sieklucka *et al.*, 2009), {[Cu_3_[*W*(CN)_8_]_2_(3-cyanopyridine)_6_]4H_2_O}_n_ (2-D), and {[Cu_3_[*W*(CN)_8_]_2_(4-cyanopyridine)_6_]8H_2_O}_n_ (2-D) (Ohkoshi *et al.*, 2003), have been reported.

The asymmetric unit of the present compound consists of a [W(CN)_8_]^3-^ anion, a [Cu1(benzimidazole)_2_]^2+^ cation and one-half of [Cu2(benzimidazole)(H_2_O)]^2+^ cation (Fig. 1). The coordination geometry of W is an 8-coordinated dodecahedron, where four CN groups of [W(CN)_8_] are bridged to Cu ions (three Cu1 and one Cu2), and other four CN groups are not bridged. The coordination geometries of the two types of Cu^II^ ions (Cu1 and Cu2) are 5-coordinated pseudo-square pyramidal. The Cu1 atom is coordinated to three nitrogen atoms of CN groups and two nitrogen atoms of benzimidazole molecules. The Cu2 atom is coordinated to two nitrogen atoms of CN groups, two nitrogen atoms of benzimidazole molecules, and an oxygen atom of an H_2_O molecule. The cyano-bridged-Cu1—W ladder chains are linked by Cu2 pillar units (Fig. 2). The benzimidazole molecules coordinated to Cu1 are aligned alternately between the layers with the intermolecular shortest distance of 3.452 (7) Å.

The field-cooled magnetization (FCM) curve at 10 Oe showed that the magnetization value gradually increased below 10 K and then drastically dropped below 7.5 K with decreasing temperature. The magnetization vs. external magnetic field (*M*-*H*) curve at 2 K showed a spin-flip transition with the critical magnetic field of 900 Oe and the saturation magnetization (*M*_s_) value of 5.2 µ_B_. This *M*_s_ value is close to the expected value of 5.0 µ_B_ for the ferromagnetic ordering of W^V^ (*S* = 1/2) ions and Cu^II^ (*S* = 1/2) ions. These FCM and *M*-*H* curves indicate that this compound is a metamagnet.

### Experimental

The title compound was prepared by reacting an aqueous solution of Cs_3_[W(CN)_8_]2H_2_O (1.2 × 10 ^-2^ mol dm^-3^) with a mixed aqueous solution of CuCl_2_2H_2_O (1.8 × 10 ^-2^ mol dm^-3^) and benzimidazole (2.0 × 10 ^-2^ mol dm^-3^) at room temperature. The prepared compound was a deep blue rod-shaped crystal. Elemental analyses: calculated for Cu_3_[*W*(CN)_8_]_2_(benzimidazole)_6_(H_2_O), Calculated: Cu, 11.20; W, 21.61; C, 40.94; H, 2.25; N, 23.05. Found: Cu, 11.32; W, 21.78; C, 40.65; H, 2.42; N, 23.15.

In the Infrared (IR) spectra, cyano stretching peaks were observed at 2206, 2201, 2186, 2183, and 2165 cm^-1^.

### Refinement

The H atoms of the benzimidazole molecules were placed in calculated positions, with C—H = 0.95 Å, and refined using a riding model, with *U*_iso_(H) = 1.2 *U*_eq_(C). The maximum and minimum residual electron density peaks were located 0.83 and 0.93 Å, respectively from the W1 atom.  A plausible water hydrogen was found in a difference map, and was refined freely.  The second water hydrogen is a symmetry equivalent of the first, because the water oxygen O1 lies on the crystallographic 2-fold.

### Crystal data

**Table:**

[Cu_3_W_2_(CN)_16_(C_7_H_6_N_2_)_6_(H_2_O)]	*F*(000) = 3300.00
*M**_r_* = 1701.46	*D*_x_ = 1.767 Mg m^−^^3^
Monoclinic, *C*2/*c*	Mo *K*α radiation, λ = 0.71075 Å
Hall symbol: -C 2yc	Cell parameters from 24759 reflections
*a* = 32.0103 (8) Å	θ = 3.0–27.5°
*b* = 10.2389 (3) Å	µ = 4.63 mm^−^^1^
*c* = 19.5533 (5) Å	*T* = 296 K
β = 93.8269 (8)°	Stick, blue
*V* = 6394.3 (3) Å^3^	0.45 × 0.12 × 0.08 mm
*Z* = 4	

### Data collection

**Table:**

Rigaku R-AXIS RAPID diffractometer	6376 reflections with *F*^2^ > 2σ(*F*^2^)
Detector resolution: 10.00 pixels mm^-1^	*R*_int_ = 0.059
ω scans	θ_max_ = 27.5°
Absorption correction: multi-scan (*ABSCOR*; Higashi, 1995)	*h* = −39→41
*T*_min_ = 0.327, *T*_max_ = 0.690	*k* = −13→13
31073 measured reflections	*l* = −25→24
7321 independent reflections	

### Refinement

**Table:**

Refinement on *F*^2^	0 restraints
*R*[*F*^2^ > 2σ(*F*^2^)] = 0.030	H atoms treated by a mixture of independent and constrained refinement
*wR*(*F*^2^) = 0.085	*w* = 1/[σ^2^(*F*_o_^2^) + (0.043*P*)^2^ + 13.526*P*] where *P* = (*F*_o_^2^ + 2*F*_c_^2^)/3
*S* = 1.02	(Δ/σ)_max_ = 0.002
7321 reflections	Δρ_max_ = 1.85 e Å^−^^3^
421 parameters	Δρ_min_ = −1.08 e Å^−^^3^

### Special details

**Table:**

Refinement. Refinement was performed using all reflections. The weighted *R*-factor (wR) and goodness of fit (*S*) are based on *F*^2^. *R*-factor (gt) are based on *F*. The threshold expression of *F*^2^ > 2.0 σ(*F*^2^) is used only for calculating *R*-factor (gt).

### Fractional atomic coordinates and isotropic or equivalent isotropic displacement parameters (Å^2^)

**Table:**

	*x*	*y*	*z*	*U*_iso_*/*U*_eq_	
W(1)	0.145909 (4)	0.677683 (13)	0.197487 (7)	0.01965 (5)	
Cu(2)	0.0000	0.49203 (8)	0.2500	0.03412 (16)	
Cu(1)	0.188606 (15)	1.17685 (4)	0.21356 (2)	0.02394 (11)	
O(1)	0.0000	0.7293 (9)	0.2500	0.122 (3)	
N(2)	0.05975 (11)	0.5193 (3)	0.23372 (18)	0.0370 (8)	
N(1)	0.17619 (12)	0.9853 (3)	0.21299 (18)	0.0346 (7)	
N(3)	0.17679 (12)	0.3708 (3)	0.21001 (18)	0.0342 (7)	
N(4)	0.24430 (12)	0.6811 (3)	0.26064 (19)	0.0339 (8)	
N(5)	0.10782 (14)	0.5221 (4)	0.0592 (2)	0.0526 (11)	
N(6)	0.06447 (17)	0.8632 (4)	0.1550 (3)	0.0742 (16)	
N(7)	0.13445 (14)	0.7107 (5)	0.36327 (19)	0.0533 (11)	
N(8)	0.19927 (14)	0.7590 (4)	0.06671 (18)	0.0495 (10)	
N(9)	0.01423 (12)	0.4524 (4)	0.34652 (19)	0.0422 (9)	
N(10)	0.04890 (15)	0.4583 (5)	0.4471 (2)	0.0610 (12)	
N(11)	0.17406 (12)	1.1776 (3)	0.31062 (18)	0.0334 (8)	
N(12)	0.17738 (19)	1.1969 (4)	0.4229 (2)	0.0611 (13)	
N(13)	0.18846 (12)	1.1756 (3)	0.11163 (18)	0.0306 (7)	
N(14)	0.16506 (13)	1.2126 (4)	0.00448 (19)	0.0410 (8)	
C(2)	0.09067 (13)	0.5704 (4)	0.22258 (19)	0.0295 (8)	
C(1)	0.16532 (12)	0.8801 (3)	0.21138 (19)	0.0276 (8)	
C(3)	0.16736 (12)	0.4779 (3)	0.20814 (18)	0.0253 (7)	
C(4)	0.21022 (13)	0.6805 (3)	0.2396 (2)	0.0276 (8)	
C(5)	0.12014 (14)	0.5790 (4)	0.1062 (2)	0.0339 (9)	
C(6)	0.09391 (15)	0.8020 (4)	0.1676 (2)	0.0399 (10)	
C(7)	0.13846 (14)	0.7019 (4)	0.3060 (2)	0.0334 (9)	
C(8)	0.18115 (14)	0.7292 (4)	0.11189 (19)	0.0329 (8)	
C(9)	0.04547 (15)	0.5048 (5)	0.3837 (2)	0.0499 (12)	
C(10)	−0.00421 (16)	0.3619 (5)	0.3883 (2)	0.0478 (11)	
C(11)	−0.03601 (18)	0.2733 (6)	0.3741 (3)	0.0692 (16)	
C(12)	−0.0462 (2)	0.1936 (7)	0.4281 (5)	0.097 (2)	
C(13)	−0.0237 (3)	0.2026 (9)	0.4940 (5)	0.111 (3)	
C(14)	0.0073 (3)	0.2874 (8)	0.5065 (3)	0.086 (2)	
C(15)	0.01723 (19)	0.3657 (6)	0.4523 (2)	0.0585 (14)	
C(16)	0.19871 (18)	1.2102 (5)	0.3653 (2)	0.0452 (11)	
C(17)	0.13574 (16)	1.1428 (4)	0.3341 (2)	0.0422 (10)	
C(18)	0.09927 (18)	1.1010 (6)	0.2988 (3)	0.0673 (16)	
C(19)	0.0650 (2)	1.0730 (8)	0.3375 (6)	0.111 (3)	
C(20)	0.0680 (3)	1.0858 (9)	0.4067 (6)	0.117 (3)	
C(21)	0.1033 (3)	1.1243 (8)	0.4433 (4)	0.104 (3)	
C(22)	0.1385 (2)	1.1537 (5)	0.4053 (3)	0.0569 (14)	
C(23)	0.15930 (14)	1.2307 (4)	0.0704 (2)	0.0365 (9)	
C(24)	0.21615 (14)	1.1184 (4)	0.0683 (2)	0.0328 (8)	
C(25)	0.25409 (15)	1.0524 (4)	0.0820 (2)	0.0426 (10)	
C(26)	0.27512 (18)	1.0159 (5)	0.0266 (3)	0.0545 (13)	
C(27)	0.2601 (2)	1.0414 (5)	−0.0407 (2)	0.0577 (14)	
C(28)	0.2242 (2)	1.1052 (5)	−0.0553 (2)	0.0526 (13)	
C(29)	0.20135 (17)	1.1435 (4)	0.0004 (2)	0.0394 (10)	
H(1)	0.025 (3)	0.784 (12)	0.233 (6)	0.20 (5)*	
H(9)	0.0633	0.5677	0.3674	0.060*	
H(10N)	0.0672	0.4809	0.4791	0.073*	
H(11)	−0.0499	0.2671	0.3309	0.083*	
H(12)	−0.0679	0.1336	0.4215	0.116*	
H(12N)	0.1873	1.2136	0.4639	0.073*	
H(13)	−0.0311	0.1475	0.5291	0.133*	
H(14)	0.0215	0.2937	0.5494	0.103*	
H(14N)	0.1489	1.2395	−0.0295	0.049*	
H(16)	0.2264	1.2378	0.3643	0.054*	
H(18)	0.0975	1.0918	0.2514	0.081*	
H(19)	0.0399	1.0454	0.3155	0.133*	
H(20)	0.0445	1.0671	0.4304	0.140*	
H(21)	0.1045	1.1312	0.4908	0.124*	
H(23)	0.1369	1.2775	0.0860	0.044*	
H(25)	0.2644	1.0343	0.1265	0.051*	
H(26)	0.3005	0.9722	0.0341	0.065*	
H(27)	0.2756	1.0132	−0.0765	0.069*	
H(28)	0.2147	1.1234	−0.1002	0.063*	

### Atomic displacement parameters (Å^2^)

**Table:**

	*U*^11^	*U*^22^	*U*^33^	*U*^12^	*U*^13^	*U*^23^
W(1)	0.02408 (10)	0.01523 (9)	0.01913 (9)	0.00006 (5)	−0.00245 (6)	−0.00061 (5)
Cu(2)	0.0216 (3)	0.0477 (4)	0.0322 (3)	0.0000	−0.0040 (2)	0.0000
Cu(1)	0.0300 (2)	0.0160 (2)	0.0255 (2)	0.00025 (17)	−0.0012 (2)	−0.00081 (16)
O(1)	0.105 (6)	0.069 (5)	0.200 (10)	0.0000	0.080 (6)	0.0000
N(2)	0.0287 (18)	0.044 (2)	0.0377 (19)	−0.0043 (15)	−0.0018 (15)	−0.0010 (15)
N(1)	0.048 (2)	0.0204 (17)	0.0353 (18)	−0.0029 (15)	0.0027 (16)	−0.0029 (13)
N(3)	0.045 (2)	0.0225 (18)	0.0347 (18)	0.0024 (15)	−0.0027 (15)	0.0004 (14)
N(4)	0.0310 (19)	0.033 (2)	0.037 (2)	−0.0025 (13)	−0.0035 (16)	0.0025 (13)
N(5)	0.070 (2)	0.050 (2)	0.035 (2)	−0.006 (2)	−0.017 (2)	−0.0103 (18)
N(6)	0.068 (3)	0.042 (2)	0.107 (4)	0.020 (2)	−0.031 (3)	−0.001 (2)
N(7)	0.055 (2)	0.079 (3)	0.0264 (19)	−0.017 (2)	0.0053 (18)	−0.0072 (18)
N(8)	0.069 (2)	0.052 (2)	0.0286 (18)	−0.014 (2)	0.0124 (19)	−0.0029 (17)
N(9)	0.0318 (19)	0.058 (2)	0.0361 (19)	−0.0047 (17)	−0.0057 (16)	0.0074 (17)
N(10)	0.058 (2)	0.083 (3)	0.040 (2)	0.004 (2)	−0.015 (2)	0.004 (2)
N(11)	0.042 (2)	0.0267 (18)	0.0315 (18)	0.0020 (14)	0.0016 (16)	−0.0003 (12)
N(12)	0.097 (4)	0.059 (3)	0.028 (2)	0.015 (2)	0.009 (2)	−0.0020 (18)
N(13)	0.0363 (19)	0.0273 (18)	0.0283 (17)	0.0036 (13)	0.0021 (15)	0.0009 (12)
N(14)	0.046 (2)	0.046 (2)	0.0304 (18)	0.0035 (18)	−0.0019 (16)	0.0012 (16)
C(2)	0.030 (2)	0.032 (2)	0.0264 (18)	−0.0022 (16)	−0.0030 (16)	−0.0040 (15)
C(1)	0.034 (2)	0.0203 (19)	0.0285 (18)	−0.0010 (15)	0.0013 (16)	−0.0035 (14)
C(3)	0.0311 (19)	0.0179 (18)	0.0262 (17)	0.0002 (14)	−0.0043 (15)	0.0004 (13)
C(4)	0.032 (2)	0.023 (2)	0.0271 (19)	−0.0037 (14)	−0.0003 (17)	0.0012 (14)
C(5)	0.041 (2)	0.030 (2)	0.030 (2)	−0.0001 (17)	−0.0059 (18)	0.0013 (16)
C(6)	0.036 (2)	0.028 (2)	0.054 (2)	0.0076 (17)	−0.010 (2)	−0.0015 (18)
C(7)	0.030 (2)	0.038 (2)	0.033 (2)	−0.0081 (17)	0.0011 (17)	−0.0037 (17)
C(8)	0.042 (2)	0.033 (2)	0.0233 (18)	−0.0021 (18)	0.0009 (17)	−0.0063 (16)
C(9)	0.037 (2)	0.073 (3)	0.038 (2)	−0.007 (2)	−0.010 (2)	0.000 (2)
C(10)	0.039 (2)	0.054 (3)	0.051 (2)	0.009 (2)	0.007 (2)	0.013 (2)
C(11)	0.046 (3)	0.060 (3)	0.102 (4)	0.002 (2)	0.009 (3)	0.013 (3)
C(12)	0.075 (5)	0.057 (4)	0.163 (9)	0.003 (3)	0.038 (5)	0.037 (4)
C(13)	0.132 (8)	0.083 (6)	0.123 (7)	0.032 (5)	0.053 (6)	0.063 (5)
C(14)	0.112 (6)	0.084 (5)	0.065 (4)	0.031 (4)	0.033 (4)	0.035 (3)
C(15)	0.062 (3)	0.067 (3)	0.047 (2)	0.024 (3)	0.006 (2)	0.011 (2)
C(16)	0.059 (3)	0.044 (2)	0.032 (2)	0.003 (2)	−0.002 (2)	−0.0059 (19)
C(17)	0.051 (2)	0.026 (2)	0.052 (2)	0.0041 (19)	0.015 (2)	−0.0012 (19)
C(18)	0.050 (3)	0.056 (3)	0.096 (4)	−0.002 (2)	0.014 (3)	−0.016 (3)
C(19)	0.058 (4)	0.086 (6)	0.197 (10)	−0.015 (3)	0.059 (5)	−0.046 (6)
C(20)	0.099 (6)	0.081 (6)	0.181 (10)	−0.019 (5)	0.093 (7)	−0.022 (6)
C(21)	0.163 (9)	0.063 (4)	0.096 (5)	0.017 (5)	0.086 (6)	0.011 (4)
C(22)	0.076 (4)	0.042 (3)	0.056 (3)	0.005 (2)	0.031 (3)	0.003 (2)
C(23)	0.043 (2)	0.035 (2)	0.031 (2)	0.0034 (19)	0.0008 (18)	−0.0015 (17)
C(24)	0.043 (2)	0.021 (2)	0.034 (2)	−0.0031 (17)	0.0031 (18)	−0.0004 (15)
C(25)	0.049 (2)	0.032 (2)	0.046 (2)	0.005 (2)	0.003 (2)	−0.0017 (19)
C(26)	0.058 (3)	0.036 (2)	0.071 (3)	0.010 (2)	0.019 (2)	−0.005 (2)
C(27)	0.081 (4)	0.042 (3)	0.055 (3)	0.000 (2)	0.032 (2)	−0.009 (2)
C(28)	0.084 (4)	0.039 (2)	0.036 (2)	−0.002 (2)	0.015 (2)	−0.004 (2)
C(29)	0.060 (3)	0.027 (2)	0.032 (2)	−0.004 (2)	0.003 (2)	0.0008 (16)

### Geometric parameters (Å, °)

**Table:**

W(1)—C(2)	2.165 (4)	C(10)—C(15)	1.388 (7)
W(1)—C(1)	2.176 (3)	C(11)—C(12)	1.390 (12)
W(1)—C(3)	2.163 (3)	C(12)—C(13)	1.436 (14)
W(1)—C(4)	2.166 (4)	C(13)—C(14)	1.329 (13)
W(1)—C(5)	2.166 (3)	C(14)—C(15)	1.383 (10)
W(1)—C(6)	2.145 (4)	C(17)—C(18)	1.384 (7)
W(1)—C(7)	2.165 (4)	C(17)—C(22)	1.394 (7)
W(1)—C(8)	2.146 (4)	C(18)—C(19)	1.403 (11)
Cu(2)—N(2)	1.980 (3)	C(19)—C(20)	1.356 (17)
Cu(2)—N(2)^i^	1.980 (3)	C(20)—C(21)	1.355 (14)
Cu(2)—N(9)	1.954 (3)	C(21)—C(22)	1.421 (12)
Cu(2)—N(9)^i^	1.954 (3)	C(24)—C(25)	1.400 (6)
Cu(1)—N(1)	2.001 (3)	C(24)—C(29)	1.403 (5)
Cu(1)—N(3)^ii^	2.022 (3)	C(25)—C(26)	1.364 (7)
Cu(1)—N(4)^iii^	2.174 (3)	C(26)—C(27)	1.396 (7)
Cu(1)—N(11)	1.984 (3)	C(27)—C(28)	1.335 (8)
Cu(1)—N(13)	1.993 (3)	C(28)—C(29)	1.408 (7)
N(2)—C(2)	1.153 (5)	O(1)—H(1)	1.05 (12)
N(1)—C(1)	1.131 (5)	O(1)—H(1)^i^	1.05 (12)
N(3)—C(3)	1.137 (5)	N(10)—H(10N)	0.860
N(4)—C(4)	1.140 (5)	N(12)—H(12N)	0.860
N(5)—C(5)	1.136 (5)	N(14)—H(14N)	0.860
N(6)—C(6)	1.145 (6)	C(9)—H(9)	0.930
N(7)—C(7)	1.139 (5)	C(11)—H(11)	0.930
N(8)—C(8)	1.131 (5)	C(12)—H(12)	0.930
N(9)—C(9)	1.312 (6)	C(13)—H(13)	0.930
N(9)—C(10)	1.392 (6)	C(14)—H(14)	0.930
N(10)—C(9)	1.326 (6)	C(16)—H(16)	0.930
N(10)—C(15)	1.396 (8)	C(18)—H(18)	0.930
N(11)—C(16)	1.327 (5)	C(19)—H(19)	0.930
N(11)—C(17)	1.385 (6)	C(20)—H(20)	0.930
N(12)—C(16)	1.362 (7)	C(21)—H(21)	0.930
N(12)—C(22)	1.345 (8)	C(23)—H(23)	0.930
N(13)—C(23)	1.318 (5)	C(25)—H(25)	0.930
N(13)—C(24)	1.394 (5)	C(26)—H(26)	0.930
N(14)—C(23)	1.328 (5)	C(27)—H(27)	0.930
N(14)—C(29)	1.367 (6)	C(28)—H(28)	0.930
C(10)—C(11)	1.378 (8)		
			
N(5)···N(10)^iv^	2.802 (5)	C(27)···H(12N)^vi^	3.506
N(7)···N(14)^v^	2.973 (5)	C(27)···H(16)^vi^	3.538
N(8)···N(12)^vi^	2.889 (5)	C(28)···H(12N)^vi^	3.500
N(8)···C(26)^vii^	3.482 (6)	H(9)···H(14)^ix^	3.551
N(8)···C(27)^vii^	3.391 (7)	H(10N)···N(5)^viii^	1.968
N(10)···N(5)^viii^	2.802 (5)	H(10N)···C(5)^viii^	2.976
N(10)···C(14)^ix^	3.324 (10)	H(10N)···C(13)^ix^	3.580
N(10)···C(15)^ix^	3.485 (7)	H(10N)···C(14)^ix^	3.389
N(12)···N(8)^v^	2.889 (5)	H(10N)···C(15)^ix^	3.472
N(12)···C(28)^v^	3.451 (7)	H(11)···C(18)^xii^	3.360
N(14)···N(7)^vi^	2.973 (5)	H(11)···H(18)^xii^	2.795
N(14)···C(26)^x^	3.453 (6)	H(11)···H(23)^xii^	3.320
N(14)···C(27)^x^	3.515 (7)	H(12)···N(6)^xii^	3.152
C(9)···C(14)^ix^	3.531 (10)	H(12)···C(23)^xii^	3.104
C(14)···N(10)^ix^	3.324 (10)	H(12)···H(18)^xii^	3.476
C(14)···C(9)^ix^	3.531 (10)	H(12)···H(20)^ix^	3.589
C(15)···N(10)^ix^	3.485 (7)	H(12)···H(21)^ix^	3.453
C(15)···C(15)^ix^	3.539 (8)	H(12)···H(23)^xii^	2.652
C(23)···C(27)^x^	3.555 (7)	H(12N)···N(8)^v^	2.040
C(24)···C(28)^x^	3.433 (7)	H(12N)···C(8)^v^	2.971
C(26)···N(8)^vii^	3.482 (6)	H(12N)···C(27)^v^	3.506
C(26)···N(14)^x^	3.453 (6)	H(12N)···C(28)^v^	3.500
C(27)···N(8)^vii^	3.391 (7)	H(13)···C(20)^ix^	2.980
C(27)···N(14)^x^	3.515 (7)	H(13)···H(20)^ix^	2.384
C(27)···C(23)^x^	3.555 (7)	H(14)···N(5)^viii^	3.340
C(27)···C(29)^x^	3.524 (7)	H(14)···N(6)^viii^	2.890
C(28)···N(12)^vi^	3.451 (7)	H(14)···N(9)^ix^	3.538
C(28)···C(24)^x^	3.433 (7)	H(14)···N(10)^ix^	3.399
C(29)···C(27)^x^	3.524 (7)	H(14)···C(5)^viii^	3.525
N(3)···H(27)^vii^	3.329	H(14)···C(6)^viii^	3.310
N(4)···H(28)^v^	3.556	H(14)···C(9)^ix^	3.307
N(5)···H(10N)^iv^	1.968	H(14)···H(9)^ix^	3.551
N(5)···H(14)^iv^	3.340	H(14N)···N(7)^vi^	2.177
N(5)···H(26)^vii^	3.558	H(14N)···C(7)^vi^	3.267
N(6)···H(12)^xi^	3.152	H(14N)···C(26)^x^	3.489
N(6)···H(14)^iv^	2.890	H(14N)···H(26)^x^	3.371
N(6)···H(21)^vi^	3.534	H(16)···C(27)^v^	3.538
N(7)···H(14N)^v^	2.177	H(16)···H(27)^v^	3.190
N(7)···H(28)^v^	3.123	H(18)···C(11)^xi^	3.566
N(8)···H(12N)^vi^	2.040	H(18)···H(11)^xi^	2.795
N(8)···H(21)^vi^	3.475	H(18)···H(12)^xi^	3.476
N(8)···H(26)^vii^	3.080	H(20)···C(13)^ix^	3.222
N(8)···H(27)^vii^	2.904	H(20)···H(12)^ix^	3.589
N(9)···H(14)^ix^	3.538	H(20)···H(13)^ix^	2.384
N(10)···H(14)^ix^	3.399	H(21)···N(6)^v^	3.534
N(12)···H(28)^v^	3.531	H(21)···N(8)^v^	3.475
N(13)···H(27)^x^	3.472	H(21)···C(6)^v^	3.561
N(14)···H(26)^x^	3.508	H(21)···C(8)^v^	3.591
C(3)···H(27)^vii^	3.255	H(21)···H(12)^ix^	3.453
C(5)···H(10N)^iv^	2.976	H(23)···C(11)^xi^	3.374
C(5)···H(14)^iv^	3.525	H(23)···C(12)^xi^	3.024
C(5)···H(27)^vii^	3.555	H(23)···H(11)^xi^	3.320
C(6)···H(14)^iv^	3.310	H(23)···H(12)^xi^	2.652
C(6)···H(21)^vi^	3.561	H(23)···H(27)^x^	3.542
C(7)···H(14N)^v^	3.267	H(26)···N(5)^vii^	3.558
C(7)···H(28)^v^	3.453	H(26)···N(8)^vii^	3.080
C(8)···H(12N)^vi^	2.971	H(26)···N(14)^x^	3.508
C(8)···H(21)^vi^	3.591	H(26)···H(14N)^x^	3.371
C(8)···H(27)^vii^	2.947	H(27)···N(3)^vii^	3.329
C(9)···H(14)^ix^	3.307	H(27)···N(8)^vii^	2.904
C(11)···H(18)^xii^	3.566	H(27)···N(13)^x^	3.472
C(11)···H(23)^xii^	3.374	H(27)···C(3)^vii^	3.255
C(12)···H(23)^xii^	3.024	H(27)···C(5)^vii^	3.555
C(13)···H(10N)^ix^	3.580	H(27)···C(8)^vii^	2.947
C(13)···H(20)^ix^	3.222	H(27)···C(16)^vi^	3.493
C(14)···H(10N)^ix^	3.389	H(27)···C(23)^x^	3.348
C(15)···H(10N)^ix^	3.472	H(27)···H(16)^vi^	3.190
C(16)···H(27)^v^	3.493	H(27)···H(23)^x^	3.542
C(16)···H(28)^v^	3.513	H(28)···N(4)^vi^	3.556
C(18)···H(11)^xi^	3.360	H(28)···N(7)^vi^	3.123
C(20)···H(13)^ix^	2.980	H(28)···N(12)^vi^	3.531
C(23)···H(12)^xi^	3.104	H(28)···C(7)^vi^	3.453
C(23)···H(27)^x^	3.348	H(28)···C(16)^vi^	3.513
C(24)···H(28)^x^	3.476	H(28)···C(24)^x^	3.476
C(25)···H(28)^x^	3.477	H(28)···C(25)^x^	3.477
C(26)···H(14N)^x^	3.489		
			
C(2)—W(1)—C(1)	133.28 (14)	N(9)—C(10)—C(11)	131.1 (5)
C(2)—W(1)—C(3)	75.96 (14)	N(9)—C(10)—C(15)	107.9 (4)
C(2)—W(1)—C(4)	133.67 (14)	C(11)—C(10)—C(15)	120.9 (5)
C(2)—W(1)—C(5)	71.25 (15)	C(10)—C(11)—C(12)	116.2 (6)
C(2)—W(1)—C(6)	74.47 (16)	C(11)—C(12)—C(13)	121.0 (7)
C(2)—W(1)—C(7)	72.00 (15)	C(12)—C(13)—C(14)	122.0 (8)
C(2)—W(1)—C(8)	141.36 (14)	C(13)—C(14)—C(15)	116.5 (7)
C(1)—W(1)—C(3)	143.38 (14)	N(10)—C(15)—C(10)	105.7 (4)
C(1)—W(1)—C(4)	71.55 (13)	N(10)—C(15)—C(14)	130.9 (5)
C(1)—W(1)—C(5)	129.57 (14)	C(10)—C(15)—C(14)	123.3 (6)
C(1)—W(1)—C(6)	71.26 (15)	N(11)—C(16)—N(12)	109.7 (4)
C(1)—W(1)—C(7)	79.50 (15)	N(11)—C(17)—C(18)	130.5 (4)
C(1)—W(1)—C(8)	72.70 (15)	N(11)—C(17)—C(22)	108.2 (4)
C(3)—W(1)—C(4)	71.85 (13)	C(18)—C(17)—C(22)	121.2 (5)
C(3)—W(1)—C(5)	74.89 (14)	C(17)—C(18)—C(19)	117.2 (6)
C(3)—W(1)—C(6)	145.36 (15)	C(18)—C(19)—C(20)	120.8 (7)
C(3)—W(1)—C(7)	93.95 (14)	C(19)—C(20)—C(21)	123.8 (9)
C(3)—W(1)—C(8)	97.32 (15)	C(20)—C(21)—C(22)	116.5 (8)
C(4)—W(1)—C(5)	128.28 (15)	N(12)—C(22)—C(17)	106.0 (5)
C(4)—W(1)—C(6)	142.77 (15)	N(12)—C(22)—C(21)	133.6 (6)
C(4)—W(1)—C(7)	77.88 (15)	C(17)—C(22)—C(21)	120.4 (6)
C(4)—W(1)—C(8)	75.95 (15)	N(13)—C(23)—N(14)	113.3 (4)
C(5)—W(1)—C(6)	78.82 (17)	N(13)—C(24)—C(25)	131.7 (3)
C(5)—W(1)—C(7)	143.17 (16)	N(13)—C(24)—C(29)	108.0 (3)
C(5)—W(1)—C(8)	70.29 (15)	C(25)—C(24)—C(29)	120.1 (4)
C(6)—W(1)—C(7)	93.73 (17)	C(24)—C(25)—C(26)	116.7 (4)
C(6)—W(1)—C(8)	94.60 (17)	C(25)—C(26)—C(27)	122.7 (5)
C(7)—W(1)—C(8)	146.55 (16)	C(26)—C(27)—C(28)	122.0 (5)
N(2)—Cu(2)—N(2)^i^	163.78 (16)	C(27)—C(28)—C(29)	117.1 (4)
N(2)—Cu(2)—N(9)	91.10 (15)	N(14)—C(29)—C(24)	105.9 (3)
N(2)—Cu(2)—N(9)^i^	92.26 (15)	N(14)—C(29)—C(28)	132.6 (4)
N(2)^i^—Cu(2)—N(9)	92.26 (15)	C(24)—C(29)—C(28)	121.4 (4)
N(2)^i^—Cu(2)—N(9)^i^	91.10 (15)	H(1)—O(1)—H(1)^i^	116 (9)
N(9)—Cu(2)—N(9)^i^	156.03 (18)	C(9)—N(10)—H(10N)	126.4
N(1)—Cu(1)—N(3)^ii^	157.80 (15)	C(15)—N(10)—H(10N)	126.4
N(1)—Cu(1)—N(4)^iii^	102.33 (14)	C(16)—N(12)—H(12N)	125.4
N(1)—Cu(1)—N(11)	87.14 (14)	C(22)—N(12)—H(12N)	125.4
N(1)—Cu(1)—N(13)	90.06 (13)	C(23)—N(14)—H(14N)	126.2
N(3)^ii^—Cu(1)—N(4)^iii^	99.67 (14)	C(29)—N(14)—H(14N)	126.2
N(3)^ii^—Cu(1)—N(11)	88.48 (14)	N(9)—C(9)—H(9)	123.7
N(3)^ii^—Cu(1)—N(13)	89.08 (13)	N(10)—C(9)—H(9)	123.7
N(4)^iii^—Cu(1)—N(11)	93.97 (14)	C(10)—C(11)—H(11)	121.9
N(4)^iii^—Cu(1)—N(13)	99.71 (14)	C(12)—C(11)—H(11)	121.9
N(11)—Cu(1)—N(13)	166.32 (15)	C(11)—C(12)—H(12)	119.5
Cu(2)—N(2)—C(2)	160.9 (3)	C(13)—C(12)—H(12)	119.5
Cu(1)—N(1)—C(1)	173.5 (3)	C(12)—C(13)—H(13)	119.0
Cu(1)^xiii^—N(3)—C(3)	175.4 (3)	C(14)—C(13)—H(13)	119.0
Cu(1)^xiv^—N(4)—C(4)	172.1 (3)	C(13)—C(14)—H(14)	121.7
Cu(2)—N(9)—C(9)	124.8 (3)	C(15)—C(14)—H(14)	121.8
Cu(2)—N(9)—C(10)	128.6 (3)	N(11)—C(16)—H(16)	125.2
C(9)—N(9)—C(10)	106.5 (3)	N(12)—C(16)—H(16)	125.2
C(9)—N(10)—C(15)	107.3 (4)	C(17)—C(18)—H(18)	121.4
Cu(1)—N(11)—C(16)	127.3 (3)	C(19)—C(18)—H(18)	121.4
Cu(1)—N(11)—C(17)	125.9 (2)	C(18)—C(19)—H(19)	119.6
C(16)—N(11)—C(17)	106.8 (3)	C(20)—C(19)—H(19)	119.6
C(16)—N(12)—C(22)	109.2 (4)	C(19)—C(20)—H(20)	118.1
Cu(1)—N(13)—C(23)	124.3 (3)	C(21)—C(20)—H(20)	118.1
Cu(1)—N(13)—C(24)	130.6 (2)	C(20)—C(21)—H(21)	121.7
C(23)—N(13)—C(24)	105.1 (3)	C(22)—C(21)—H(21)	121.7
C(23)—N(14)—C(29)	107.6 (3)	N(13)—C(23)—H(23)	123.3
W(1)—C(2)—N(2)	175.6 (3)	N(14)—C(23)—H(23)	123.4
W(1)—C(1)—N(1)	174.2 (3)	C(24)—C(25)—H(25)	121.7
W(1)—C(3)—N(3)	175.3 (3)	C(26)—C(25)—H(25)	121.7
W(1)—C(4)—N(4)	178.7 (3)	C(25)—C(26)—H(26)	118.7
W(1)—C(5)—N(5)	176.6 (3)	C(27)—C(26)—H(26)	118.7
W(1)—C(6)—N(6)	174.9 (4)	C(26)—C(27)—H(27)	119.0
W(1)—C(7)—N(7)	177.9 (4)	C(28)—C(27)—H(27)	119.0
W(1)—C(8)—N(8)	178.4 (4)	C(27)—C(28)—H(28)	121.4
N(9)—C(9)—N(10)	112.6 (4)	C(29)—C(28)—H(28)	121.4
			
C(2)—W(1)—C(1)—N(1)	−131 (3)	N(13)—Cu(1)—N(1)—C(1)	−82 (3)
C(1)—W(1)—C(2)—N(2)	53 (4)	N(3)^ii^—Cu(1)—N(4)^iii^—C(4)^iii^	100 (2)
C(2)—W(1)—C(3)—N(3)	64 (4)	N(4)^iii^—Cu(1)—N(3)^ii^—C(3)^ii^	−165 (4)
C(3)—W(1)—C(2)—N(2)	−153 (4)	N(3)^ii^—Cu(1)—N(11)—C(16)	−82.6 (3)
C(2)—W(1)—C(4)—N(4)	115 (14)	N(3)^ii^—Cu(1)—N(11)—C(17)	98.0 (3)
C(4)—W(1)—C(2)—N(2)	160 (4)	N(11)—Cu(1)—N(3)^ii^—C(3)^ii^	−72 (4)
C(2)—W(1)—C(5)—N(5)	−89 (6)	N(3)^ii^—Cu(1)—N(13)—C(23)	−47.8 (3)
C(5)—W(1)—C(2)—N(2)	−74 (4)	N(3)^ii^—Cu(1)—N(13)—C(24)	133.9 (3)
C(2)—W(1)—C(6)—N(6)	22 (5)	N(13)—Cu(1)—N(3)^ii^—C(3)^ii^	95 (4)
C(6)—W(1)—C(2)—N(2)	9(4)	N(4)^iii^—Cu(1)—N(11)—C(16)	17.0 (3)
C(2)—W(1)—C(7)—N(7)	51 (11)	N(4)^iii^—Cu(1)—N(11)—C(17)	−162.4 (3)
C(7)—W(1)—C(2)—N(2)	108 (4)	N(11)—Cu(1)—N(4)^iii^—C(4)^iii^	11 (2)
C(2)—W(1)—C(8)—N(8)	98 (13)	N(4)^iii^—Cu(1)—N(13)—C(23)	−147.5 (3)
C(8)—W(1)—C(2)—N(2)	−68 (4)	N(4)^iii^—Cu(1)—N(13)—C(24)	34.3 (3)
C(1)—W(1)—C(3)—N(3)	−148 (4)	N(13)—Cu(1)—N(4)^iii^—C(4)^iii^	−169 (2)
C(3)—W(1)—C(1)—N(1)	93 (3)	N(11)—Cu(1)—N(13)—C(23)	32.0 (7)
C(1)—W(1)—C(4)—N(4)	−112 (14)	N(11)—Cu(1)—N(13)—C(24)	−146.3 (5)
C(4)—W(1)—C(1)—N(1)	95 (3)	N(13)—Cu(1)—N(11)—C(16)	−162.4 (5)
C(1)—W(1)—C(5)—N(5)	140 (6)	N(13)—Cu(1)—N(11)—C(17)	18.1 (7)
C(5)—W(1)—C(1)—N(1)	−30 (3)	Cu(2)—N(2)—C(2)—W(1)	−28 (5)
C(1)—W(1)—C(6)—N(6)	−126 (5)	Cu(1)—N(1)—C(1)—W(1)	95 (4)
C(6)—W(1)—C(1)—N(1)	−87 (3)	Cu(1)^xiii^—N(3)—C(3)—W(1)	−53 (7)
C(1)—W(1)—C(7)—N(7)	−166 (11)	Cu(1)^xiv^—N(4)—C(4)—W(1)	11 (16)
C(7)—W(1)—C(1)—N(1)	176 (3)	Cu(2)—N(9)—C(9)—N(10)	−178.3 (3)
C(1)—W(1)—C(8)—N(8)	−42 (13)	Cu(2)—N(9)—C(10)—C(11)	2.3 (8)
C(8)—W(1)—C(1)—N(1)	15 (3)	Cu(2)—N(9)—C(10)—C(15)	178.3 (3)
C(3)—W(1)—C(4)—N(4)	66 (14)	C(9)—N(9)—C(10)—C(11)	−174.8 (6)
C(4)—W(1)—C(3)—N(3)	−150 (4)	C(9)—N(9)—C(10)—C(15)	1.2 (6)
C(3)—W(1)—C(5)—N(5)	−9(6)	C(10)—N(9)—C(9)—N(10)	−1.1 (6)
C(5)—W(1)—C(3)—N(3)	−10 (4)	C(9)—N(10)—C(15)—C(10)	0.3 (6)
C(3)—W(1)—C(6)—N(6)	54 (5)	C(9)—N(10)—C(15)—C(14)	178.9 (7)
C(6)—W(1)—C(3)—N(3)	31 (4)	C(15)—N(10)—C(9)—N(9)	0.5 (6)
C(3)—W(1)—C(7)—N(7)	−22 (11)	Cu(1)—N(11)—C(16)—N(12)	−179.4 (3)
C(7)—W(1)—C(3)—N(3)	134 (4)	Cu(1)—N(11)—C(17)—C(18)	−0.3 (5)
C(3)—W(1)—C(8)—N(8)	174 (13)	Cu(1)—N(11)—C(17)—C(22)	178.7 (3)
C(8)—W(1)—C(3)—N(3)	−78 (4)	C(16)—N(11)—C(17)—C(18)	−179.8 (3)
C(4)—W(1)—C(5)—N(5)	42 (7)	C(16)—N(11)—C(17)—C(22)	−0.9 (5)
C(5)—W(1)—C(4)—N(4)	14 (14)	C(17)—N(11)—C(16)—N(12)	0.1 (4)
C(4)—W(1)—C(6)—N(6)	−123 (5)	C(16)—N(12)—C(22)—C(17)	−1.3 (6)
C(6)—W(1)—C(4)—N(4)	−115 (14)	C(16)—N(12)—C(22)—C(21)	−179.3 (7)
C(4)—W(1)—C(7)—N(7)	−93 (11)	C(22)—N(12)—C(16)—N(11)	0.7 (6)
C(7)—W(1)—C(4)—N(4)	165 (14)	Cu(1)—N(13)—C(23)—N(14)	−177.5 (3)
C(4)—W(1)—C(8)—N(8)	−117 (13)	Cu(1)—N(13)—C(24)—C(25)	−5.3 (6)
C(8)—W(1)—C(4)—N(4)	−36 (14)	Cu(1)—N(13)—C(24)—C(29)	178.5 (3)
C(5)—W(1)—C(6)—N(6)	95 (5)	C(23)—N(13)—C(24)—C(25)	176.2 (4)
C(6)—W(1)—C(5)—N(5)	−166 (6)	C(23)—N(13)—C(24)—C(29)	−0.0 (3)
C(5)—W(1)—C(7)—N(7)	48 (11)	C(24)—N(13)—C(23)—N(14)	1.1 (5)
C(7)—W(1)—C(5)—N(5)	−85 (6)	C(23)—N(14)—C(29)—C(24)	1.6 (5)
C(5)—W(1)—C(8)—N(8)	103 (13)	C(23)—N(14)—C(29)—C(28)	−174.7 (5)
C(8)—W(1)—C(5)—N(5)	95 (6)	C(29)—N(14)—C(23)—N(13)	−1.8 (5)
C(6)—W(1)—C(7)—N(7)	124 (11)	N(9)—C(10)—C(11)—C(12)	178.2 (6)
C(7)—W(1)—C(6)—N(6)	−48 (5)	N(9)—C(10)—C(15)—N(10)	−0.9 (6)
C(6)—W(1)—C(8)—N(8)	27 (13)	N(9)—C(10)—C(15)—C(14)	−179.6 (6)
C(8)—W(1)—C(6)—N(6)	164 (5)	C(11)—C(10)—C(15)—N(10)	175.5 (5)
C(7)—W(1)—C(8)—N(8)	−77 (13)	C(11)—C(10)—C(15)—C(14)	−3.2 (10)
C(8)—W(1)—C(7)—N(7)	−132 (11)	C(15)—C(10)—C(11)—C(12)	2.7 (9)
N(2)—Cu(2)—N(2)^i^—C(2)^i^	−7.9 (13)	C(10)—C(11)—C(12)—C(13)	−1.5 (11)
N(2)^i^—Cu(2)—N(2)—C(2)	−7.9 (13)	C(11)—C(12)—C(13)—C(14)	0.6 (13)
N(2)—Cu(2)—N(9)—C(9)	33.0 (4)	C(12)—C(13)—C(14)—C(15)	−0.8 (14)
N(2)—Cu(2)—N(9)—C(10)	−143.5 (4)	C(13)—C(14)—C(15)—N(10)	−176.3 (7)
N(9)—Cu(2)—N(2)—C(2)	−109.8 (10)	C(13)—C(14)—C(15)—C(10)	2.1 (12)
N(2)—Cu(2)—N(9)^i^—C(9)^i^	−131.1 (4)	N(11)—C(17)—C(18)—C(19)	−179.7 (5)
N(2)—Cu(2)—N(9)^i^—C(10)^i^	52.3 (4)	N(11)—C(17)—C(22)—N(12)	1.3 (5)
N(9)^i^—Cu(2)—N(2)—C(2)	93.9 (10)	N(11)—C(17)—C(22)—C(21)	179.7 (5)
N(2)^i^—Cu(2)—N(9)—C(9)	−131.1 (4)	C(18)—C(17)—C(22)—N(12)	−179.6 (4)
N(2)^i^—Cu(2)—N(9)—C(10)	52.3 (4)	C(18)—C(17)—C(22)—C(21)	−1.3 (8)
N(9)—Cu(2)—N(2)^i^—C(2)^i^	93.9 (10)	C(22)—C(17)—C(18)—C(19)	1.4 (8)
N(2)^i^—Cu(2)—N(9)^i^—C(9)^i^	33.0 (4)	C(17)—C(18)—C(19)—C(20)	−0.5 (10)
N(2)^i^—Cu(2)—N(9)^i^—C(10)^i^	−143.5 (4)	C(18)—C(19)—C(20)—C(21)	−0.6 (11)
N(9)^i^—Cu(2)—N(2)^i^—C(2)^i^	−109.8 (10)	C(19)—C(20)—C(21)—C(22)	0.8 (13)
N(9)—Cu(2)—N(9)^i^—C(9)^i^	131.1 (4)	C(20)—C(21)—C(22)—N(12)	178.0 (7)
N(9)—Cu(2)—N(9)^i^—C(10)^i^	−45.5 (6)	C(20)—C(21)—C(22)—C(17)	0.1 (8)
N(9)^i^—Cu(2)—N(9)—C(9)	131.1 (4)	N(13)—C(24)—C(25)—C(26)	−175.5 (4)
N(9)^i^—Cu(2)—N(9)—C(10)	−45.5 (6)	N(13)—C(24)—C(29)—N(14)	−1.0 (4)
N(1)—Cu(1)—N(3)^ii^—C(3)^ii^	7(4)	N(13)—C(24)—C(29)—C(28)	175.8 (4)
N(3)^ii^—Cu(1)—N(1)—C(1)	5(3)	C(25)—C(24)—C(29)—N(14)	−177.8 (4)
N(1)—Cu(1)—N(4)^iii^—C(4)^iii^	−77 (2)	C(25)—C(24)—C(29)—C(28)	−0.9 (6)
N(4)^iii^—Cu(1)—N(1)—C(1)	178 (2)	C(29)—C(24)—C(25)—C(26)	0.3 (6)
N(1)—Cu(1)—N(11)—C(16)	119.2 (3)	C(24)—C(25)—C(26)—C(27)	−0.3 (6)
N(1)—Cu(1)—N(11)—C(17)	−60.3 (3)	C(25)—C(26)—C(27)—C(28)	0.9 (8)
N(11)—Cu(1)—N(1)—C(1)	84 (2)	C(26)—C(27)—C(28)—C(29)	−1.4 (8)
N(1)—Cu(1)—N(13)—C(23)	110.0 (3)	C(27)—C(28)—C(29)—N(14)	177.3 (5)
N(1)—Cu(1)—N(13)—C(24)	−68.3 (3)	C(27)—C(28)—C(29)—C(24)	1.5 (7)

Symmetry codes: (i) −*x*, *y*, −*z*+1/2; (ii) *x*, *y*+1, *z*; (iii) −*x*+1/2, *y*+1/2, −*z*+1/2; (iv) *x*, −*y*+1, *z*−1/2; (v) *x*, −*y*+2, *z*+1/2; (vi) *x*, −*y*+2, *z*−1/2; (vii) −*x*+1/2, −*y*+3/2, −*z*; (viii) *x*, −*y*+1, *z*+1/2; (ix) −*x*, −*y*+1, −*z*+1; (x) −*x*+1/2, −*y*+5/2, −*z*; (xi) −*x*, *y*+1, −*z*+1/2; (xii) −*x*, *y*−1, −*z*+1/2; (xiii) *x*, *y*−1, *z*; (xiv) −*x*+1/2, *y*−1/2, −*z*+1/2.

### Hydrogen-bond geometry (Å, °)

**Table:**

*D*—H···*A*	*D*—H	H···*A*	*D*···*A*	*D*—H···*A*
O(1)—H(1)···N(6)	1.05 (12)	2.20 (12)	3.178 (7)	154 (10)
N(10)—H(10N)···N(5)^viii^	0.86	1.97	2.802 (5)	163
N(12)—H(12N)···N(8)^v^	0.86	2.04	2.888 (5)	169
N(14)—H(14N)···N(7)^vi^	0.86	2.18	2.973 (5)	154

Symmetry codes: (viii) *x*, −*y*+1, *z*+1/2; (v) *x*, −*y*+2, *z*+1/2; (vi) *x*, −*y*+2, *z*−1/2.

## References

[bb1] Catala, L., Mathonière, C., Gloter, A., Stephan, O., Gacoin, T., Boilot, J.-P. & Mallah, T. (2005). *Chem. Commun.* pp. 746–748. 10.1039/b415157g 15685324

[bb2] Farrugia, L. J. (1997). *J. Appl. Cryst.* **30**, 565.

[bb3] Garde, R., Desplanches, C., Bleuzen, A., Veillet, P. & Verdaguer, M. (1999). *Mol. Cryst. Liq. Cryst.* **334**, 587–595.

[bb4] Herrera, J. M., Franz, P., Podgajny, R., Pilkington, M., Biner, M., Decurtins, S., Stoeckli-Evans, H., Neels, A., Garde, R., Dromzée, Y., Julve, M., Sieklucka, B., Hashimoto, K., Ohkoshi, S. & Verdaguer, M. (2008). *C. R. Chim.* **11**, 1192–1199.

[bb5] Herrera, J. M., Marvaud, V., Verdaguer, M., Marrot, J., Kalisz, M. & Mathonière, C. (2004). *Angew. Chem. Int. Ed.* **43**, 5468–5471. 10.1002/anie.200460387 15372638

[bb6] Higashi, T. (1995). *ABSCOR.* Rigaku Corporation, Tokyo, Japan.

[bb7] Kaneko, S., Tsunobuchi, Y., Sakurai, S. & Ohkoshi, S. (2007). *Chem. Phys. Lett.* **446**, 292–296.

[bb8] Kosaka, W., Imoto, K., Tsunobuchi, Y. & Ohkoshi, S. (2009). *Inorg. Chem.* **48**, 4604–4606. 10.1021/ic9001576 19378953

[bb9] Leipoldt, J. G., Basson, S. S. & Roodt, A. (1994). *Adv. Inorg. Chem.* **40**, 241–322.

[bb10] Momma, K. & Izumi, F. (2006). *IUCr Commission on Crystallographic Computing Newsletter*, **130**, 106–119.

[bb11] Ohkoshi, S., Arimoto, Y., Hozumi, T., Seino, H., Mizobe, Y. & Hashimoto, K. (2003). *Chem. Commun.* pp. 2772–2773. 10.1039/b310456g 14651098

[bb12] Ohkoshi, S., Hamada, Y., Matsuda, T., Tsunobuchi, Y. & Tokoro, H. (2008). *Chem. Mater.* **20**, 3048–3054.

[bb13] Ohkoshi, S., Ikeda, S., Hozumi, T., Kashiwagi, T. & Hashimoto, K. (2006). *J. Am. Chem. Soc.* **128**, 5320–5321. 10.1021/ja060510e 16620085

[bb14] Ohkoshi, S., Tsunobuchi, Y., Takahashi, H., Hozumi, T., Shiro, M. & Hashimoto, K. (2007). *J. Am. Chem. Soc.* **129**, 3084–3085. 10.1021/ja069353+ 17311388

[bb15] Podgajny, R., Korzeniak, T., Bałanda, M., Wasiutyński, T., Errington, W., Kemp, J. T., Alcock, W. N. & Sieklucka, B. (2002). *Chem. Commun.* pp. 1138–1139. 10.1039/b202810g 12122703

[bb16] Rigaku (1998). *PROCESS-AUTO.* Rigaku Corporation, Tokyo, Japan.

[bb17] Rigaku (2007). *CrystalStructure.* Rigaku Corporation, Tokyo, Japan, and Rigaku Americas, The Woodlands, Texas, USA.

[bb18] Sheldrick, G. M. (2008). *Acta Cryst.* A**64**, 112–122. 10.1107/S0108767307043930 18156677

[bb19] Sieklucka, B., Podgajny, R., Pinkowicz, D., Nowicka, B., Korzeniak, T., Bałanda, M., Wasiutyński, T., Pełka, R., Makarewicz, M., Czapa, M., Rams, M., Gaweł, B. & Łasocha, W. (2009). *CrystEngComm*, **11**, 2032–2039.

[bb20] Zhong, Z. J., Seino, H., Mizobe, Y., Hidai, M., Verdaguer, M., Ohkoshi, S. & Hashimoto, K. (2000). *Inorg. Chem.* **39**, 5095–5101. 10.1021/ic0005994 11233207

